# Parathyroid Hormone Measurement in Chronic Kidney Disease: From Basics to Clinical Implications

**DOI:** 10.1155/2019/5496710

**Published:** 2019-09-17

**Authors:** Kittrawee Kritmetapak, Chatlert Pongchaiyakul

**Affiliations:** ^1^Division of Nephrology, Department of Medicine, Faculty of Medicine, Khon Kaen University, Khon Kaen 40002, Thailand; ^2^Division of Endocrinology and Metabolism, Department of Medicine, Faculty of Medicine, Khon Kaen University, Khon Kaen 40002, Thailand

## Abstract

Accurate measurement of parathyroid hormone (PTH) is crucial for therapeutic decision-making in patients with chronic kidney disease-mineral and bone disorder (CKD-MBD). The second-generation PTH assays, often referred to as “intact PTH” assays, are the current standard and most available assays in clinical practice. However, intact PTH assays measure both full-length biologically active PTH and heterogeneous PTH fragments in the circulation, providing the equivocal value of PTH measurement in patients with CKD-MBD. Due to the variability of PTH assays, preanalytical sample errors, and the phenomenon of end-organ PTH hyporesponsiveness, current CKD-MBD guidelines recommend a wide range for serum PTH targets (2–9 the upper normal limit of the intact PTH assay) in dialysis patients to diminish the risk of developing adynamic bone disease. Nevertheless, a sizeable proportion of CKD patients still experience renal osteodystrophy despite having serum PTH levels within the recommended range. The primary cause of this inconsistency is the analytical interference of various PTH fragments and oxidized PTH forms that considerably accumulate in CKD patients. Therefore, a new mass spectrometry-based assay, which is capable of specifically measuring the whole spectra of PTH fragments, can potentially improve diagnostic accuracy for renal osteodystrophy. However, the effects of different PTH fragments on bone metabolism, vascular calcification, and mortality in CKD patients warrant further research.

## 1. Introduction

Chronic kidney disease-mineral and bone disorder (CKD-MBD), characterized by deranged metabolism of calcium, phosphate, parathyroid hormone (PTH), fibroblast growth factor 23 (FGF23), and vitamin D; bone abnormalities, formerly known as “renal osteodystrophy”; and vascular calcification, is a well-established complication of CKD [1]. CKD-MBD contributes to the large burden of cardiovascular disease, which is the leading cause of death in patients with CKD [2]. The complex pathophysiology of CKD-MBD involves a number of feedback loops between the kidney, parathyroid glands, bone, intestine, and vasculature, and usually commences early in the course of CKD prior to the onset of clinically detectable abnormalities in serum calcium, phosphate, PTH, and vitamin D levels [3–6]. Previous studies demonstrated that patients with CKD have decreased renal Klotho expression as early as CKD G1. As CKD progresses, Klotho concentration continues to decline, causing FGF23 resistance and, therefore, leading to marked increases in serum FGF23 (CKD G2–G5) and PTH levels (CKD G3a–G5), as well as decreases in serum 25-hydroxyvitamin D (25(OH)D) levels (CKD G2–G5) [7–9]. However, hypocalcemia and hyperphosphatemia are usually observed in CKD G4–G5.

PTH reflects the function of the parathyroid gland and also primarily takes part in the metabolism of calcium, phosphate, FGF23, and vitamin D. Nevertheless, lack of standardization, insufficient validity, and nonspecific measurement of various PTH fragments among current PTH assays are mainly accountable for the wide range of the serum PTH targets in dialysis patients, approximately 2 to 9 times the upper normal limit for the assay, suggested by Kidney Disease: Improving Global Outcomes (KDIGO) in 2017. In this review, we provide a summary of the PTH metabolism and pathophysiology underlying PTH derangements in CKD-MBD as well as clinical considerations for PTH measurement from the past to the present.

## 2. Parathyroid Hormone (PTH) Physiology and Metabolism

PTH is the primary calcium- and phosphate-regulating hormone produced by chief cells in the parathyroid glands. PTH is initially synthesized as a 115-amino acid polypeptide called preproPTH, which is proteolytically cleaved within the rough endoplasmic reticulum at the amino-terminal (N-terminal) part first to proPTH (90 amino acids) and subsequently to PTH (84 amino acids) in the Golgi complex. PTH is the major storage, secreted, and biologically active form of the hormone, and it has a molecular weight of approximately 9500 Dalton [10, 11]. Enhanced PTH secretion occurs in response to hypocalcemia, hyperphosphatemia, and/or a decrease in serum 1,25-dihydroxyvitamin D (1,25(OH)_2_D) level, whereas high serum levels of calcium, calcitriol, or FGF23 suppress PTH secretion. The extracellular concentration of ionized calcium is the most essential determinant of the minute-to-minute oscillatory secretion of PTH, which tends to be blunted in CKD patients [12]. Once secreted, PTH is rapidly cleared from plasma through cellular uptake principally by the liver and kidneys, where PTH undergoes intracellular proteolysis into active amino- and inactive carboxyl-terminal PTH fragments. The fate of these two types of PTH fragments is different: the N-terminal PTH fragments are rapidly degraded in situ by the liver and kidney, whereas the carboxyl-terminal (C-terminal) PTH fragments are mainly released into the blood and eventually excreted by the kidney [13].

PTH exerts the biological effects via the interaction of its 34 N-terminal amino acids with the G protein-coupled type 1 PTH/PTH-related peptide (PTHrP) receptor (PTHR1), then promotes generation of intracellular cyclic adenosine monophosphate (cAMP), and leads to activation of protein kinase A and C pathways in renal tubular cells, osteoblasts, and osteocytes [14]. In contrast, PTH 2 receptor (PTHR2) is activated selectively by PTH but not by PTHrP, and it is abundant in the brain, pancreas, testes, and placenta [15]; however, the real physiological significance of PTHR2 is still unknown. PTH and N-terminal PTH fragments have very short plasma half-lives between 2 and 4 minutes, whereas the C-terminal PTH fragments have a half-life of several hours and even longer in patients with CKD due to decreased renal clearance.

## 3. Parathyroid Hormone- (PTH-) Related Derangements in CKD-MBD

**Figure 1:**
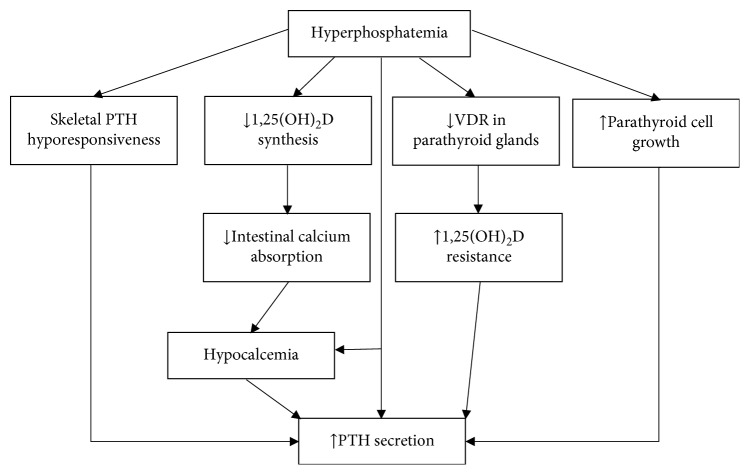
Mechanism of phosphate-induced secondary hyperparathyroidism. PTH, parathyroid hormone; VDR, vitamin D receptor.

With progression of CKD, phosphate is retained due to decreased urinary phosphate excretion. However, hyperphosphatemia usually does not become evident before CKD G4. Until then, compensatory increases in circulating FGF23 and PTH result in increased phosphaturia, hence maintaining serum phosphate levels in the normal range [16, 17]. The mechanisms how phosphate retention contributes to the development of secondary hyperparathyroidism are multifactorial (Figure 1) [18, 19], including (1) the induction of hypocalcemia, (2) diminished renal production of 1,25(OH)_2_D by inhibiting 1-alpha-hydroxylase activity, (3) increased PTH gene expression by reducing PTH mRNA degradation, (4) direct stimulation of parathyroid growth, and (5) stimulation of FGF23 production in bone.

FGF23 is a 32,000 Da phosphaturic hormone that is secreted primarily by osteocytes and osteoblasts in response to increased serum calcium, phosphate, PTH, or 1,25(OH)_2_D. FGF23 plays a vital role as a regulator of phosphate homeostasis through inhibition of sodium-phosphate cotransporter and 1-alpha-hydroxylase activity, resulting in reduction of proximal tubular phosphate reabsorption and 1,25(OH)_2_D production, respectively. In addition, FGF23 acts directly on the parathyroid glands and has inhibitory effects on PTH secretion and parathyroid growth [20–23]. FGF23 and PTH mutually regulate each other in a negative feedback loop, where PTH stimulates FGF23 synthesis and FGF23 in turn inhibits PTH production [22].

1,25(OH)_2_D is primarily synthesized by the proximal tubule through the 1-alpha-hydroxylation of 25(OH)D, which is mediated by the 1-alpha-hydroxylase. PTH increases the 1,25(OH)_2_D production by enhancing 1-alpha-hydroxylase activity, whereas phosphate and FGF23 have the opposite effect in this regard. In turn, 1,25(OH)_2_D suppresses the production of PTH by either directly decreasing the PTH gene transcription or indirectly increasing the intestinal calcium absorption and stimulating the CaSR expression in the parathyroid glands [24, 25]. Enhanced PTH release occurs in response to hypocalcemia, hyperphosphatemia, and low 1,25(OH)_2_D level, whereas high serum levels of calcium, 1,25(OH)_2_D, or FGF23 suppress PTH production. The predominant effects of FGF23 on the stimulation of PTH production appear to be indirect as a result of the potent effect of FGF23 to reduce 1,25(OH)_2_D synthesis [26–28].

There are also intrinsic abnormalities in parathyroid gland structure and function in patients with CKD. Resected parathyroid glands from CKD patients with severe hyperparathyroidism have nodular areas throughout the gland, which represent monoclonal expansions of parathyroid cells. Within the parathyroid nodules, the expression of calcium-sensing receptors and vitamin D receptors is decreased, leading to an increased set-point for calcium- and vitamin D-regulated PTH secretion [24, 29]. There is also evidence supporting the reduced Klotho and FGF receptor expression in hyperplastic parathyroid glands, particularly in cases with extremely high FGF23 levels, leading to FGF23 resistance and secondary hyperparathyroidism [30]. Furthermore, end-organ hyporesponsiveness to PTH, formerly known as skeletal PTH resistance, has long been recognized in CKD. The resultant decrease in serum calcium levels stimulates PTH secretion and contributes to the pathogenesis of secondary hyperparathyroidism. The pathophysiologic factors involved in the end-organ hyporesponsiveness to PTH include downregulation or desensitization of PTHR1, oxidative modification of PTH structure, vitamin D deficiency, phosphate retention, and increase in levels of certain uremic toxins such as indoxyl sulfate, osteoprotegerin, and sclerostin [31–34]. Additionally, the accumulation of C-terminal PTH fragments may serve to antagonize the calcemic actions of PTH, not only by acting at a C-terminal PTH receptor on osteoblasts and osteocytes but also by reducing PTHR1 expression [35, 36].

Skeletal hyporesponsiveness to PTH in early CKD stages could link to a relatively high prevalence of low turnover bone disease, especially adynamic bone disease. Peritoneal dialysis patients have higher serum levels of circulating C-terminal PTH fragments than hemodialysis patients, and this may partially explain why adynamic bone disease is more common in peritoneal dialysis patients [37]. Moreover, in advanced stages of CKD, the constantly increasing PTH stimulation of PTHR1 ultimately prevails over the skeletal hyporesponsiveness to the action of PTH, and high turnover bone diseases (osteitis fibrosa or mixed uremic osteodystrophy) develop.

## 4. Evolution of PTH Assays

### 4.1. First-Generation PTH Assay

PTH was originally determined by the C-terminal radioimmunoassay (RIA) in 1963 [10]. The first-generation PTH assay used a polyclonal PTH antibody directed against diverse epitopes that was predominantly within the mid- or C-terminal part of the PTH structure. Hence, the first-generation PTH assay detected nonspecifically both PTH (1-84) and a number of C-terminal PTH fragments lacking an N-terminal part [10, 38]. In humans, C-terminal PTH fragments typically started their structure at amino acid position 34, 37, 38, or 45 and thus completely lacked the biologically active N-terminal part of PTH (1-84) [39, 40]. It is uncertain if all C-terminal PTH fragments are intact up to position 84. C-terminal PTH fragments originate from calcium-dependent parathyroid gland secretion and calcium-independent peripheral PTH metabolism, mainly by Kupffer cells in the liver, and they are eventually eliminated by the kidney [41–43].

Intact PTH (1-84) and C-terminal PTH fragments, respectively, represented 20% and 80% of the circulating PTH measured by the first-generation PTH assay [44]. The proportion of measured C-terminal PTH fragment increased up to 95% of circulating PTH in patients with CKD [45, 46]. As a result of inadequate sensitivity and dismal specificity caused by substantial cross-reactivity with biologically inactive PTH fragments, the RIA has thereafter been replaced by the second-generation PTH assay using the sandwich immunoassay technique such as chemiluminescence immunoassay (CLIA), enzyme-linked immunosorbent assay (ELISA), and immunoradiometric assay (IRMA).

### 4.2. Second-Generation PTH Assay

The second-generation PTH assay, erroneously called the intact PTH assay, was developed in 1987 and it is currently the most common assay for measuring PTH level. The intact PTH assay used double PTH antibody comprising a solid-phase capture antibody (coated antibody) against the C-terminal part of the PTH (amino acid positions 39-84) and a detection antibody (labelled antibody) against the N-terminal part of the PTH (amino acid positions 12-18, 13-24, or 26-32). Therefore, the intact PTH assay actually recognized both the full-length biologically active PTH (1-84) and large C-terminal PTH fragments with a partially preserved N-terminal part, called “non-(1-84) PTH fragments” or “N-terminal truncated PTH fragments.”

Non-(1-84) PTH fragments represent approximately 10% of circulating C-terminal PTH fragments in patients with CKD [47]. The structure of non-(1-84) PTH fragments usually start at amino acid position 4, 7, 8, 10, or 15 with the major fragment presumably starting at position 7, called PTH (7-84) [48]. However, the actual structure of PTH (7-84) has not been directly demonstrated by Edman sequencing or mass spectrometry [49]. Compared with the PTH (1-84), non-(1-84) PTH fragments have a longer half-life and accumulate in CKD patients due to decreased renal clearance and increased PTH fragment secretion from the parathyroid glands [50]. Non-(1-84) PTH fragments accounted for 20% of the circulating PTH measured by the second-generation PTH assay but only 5% of the circulating PTH measured by the first-generation PTH assay [51]. In patients with CKD, non-(1-84) PTH fragments increase up to 50% of the circulating PTH levels measured by the second-generation PTH assay [45, 48, 52].

Non-(1-84) PTH fragment, specifically PTH (7-84), does not bind avidly to PTHR1; however, it apparently exerts some of its biological effects through a C-terminal PTH receptor on osteoblasts, osteocytes, and renal tubular cells [27, 46]. Synthetic PTH (7-84) inhibits osteoblast differentiation, induces osteocyte apoptosis, inhibits bone resorption, and reduces 1,25(OH)_2_D synthesis in the kidney [46, 53]. In an in vivo study, synthetic PTH (7-84) has been shown to have hypocalcemic effects, which was demonstrated by its ability to suppress the hypercalcemic effects of PTH (1-34) and PTH (1-84) [27, 52, 54, 55]. Therefore, PTH (7-84) exerts the antagonistic effect to PTH (1-84) on bone turnover rate and may contribute to end-organ hyporesponsiveness to PTH in patients with CKD [27, 36, 56]. Hypocalcemia promotes PTH (1-84) release but decreases non-(1-84) PTH fragment secretion, which maximizes the hypercalcemic effect of PTH (1-84) through PTHR1. Indeed, hypercalcemia not only decreases PTH (1-84) secretion but also increases non-(1-84) PTH fragment secretion, which maximizes the hypocalcemic effect of non-(1-84) PTH fragment through the C-terminal PTH receptor [57]. Elevated serum phosphate levels directly stimulate PTH synthesis but reduce non-(1-84) PTH fragment secretion that are expected to maximize the urinary phosphate excretion during hyperphosphatemia [58, 59]. Previous literatures and recommendations from the 2017 KDIGO CKD-MBD guideline were based on the second-generation Allegro PTH assay from Nichols, which is not currently available [60, 61]. Moreover, the different intact PTH assays measure different types and amounts of the circulating PTH fragments, yielding variability and inconsistent results among the PTH measurements [62]. Therefore, the third-generation PTH assay has been developed in order to improve diagnostic accuracy.

### 4.3. Third-Generation PTH Assay

The third-generation PTH assay, also known as the “bioactive,” “biointact,” or “whole” PTH (1-84) assay, has become available since 1999 by using a similar capture antibody in the intact PTH assay but uses a different detection antibody directed against the most proximal end of the N-terminus (amino acid positions 1-4) of the PTH structure and, therefore, was initially presumed to measure solely the biologically active PTH (1-84), but not non-PTH (1-84) fragments [63–65]. However, subsequent studies proved that the bioactive PTH assay not only reacted with the full-length PTH (1-84) but also cross-reacted to an N-terminal PTH form that is not detected by most intact PTH assays, depending on the epitopes of PTH molecule that the assays recognize [45, 52]. The inability of the intact PTH assay to detect the N-terminal PTH form is apparently due to a posttranslational phosphorylation at serine position 17 of the PTH (1-84) structure and a reduction in binding affinity for antibody detection. In individuals with normal renal function, the N-terminal PTH form accounts for 4–8% of circulating PTH measured by the bioactive PTH assay, but increases to 15% in patients with CKD [63, 64]. Moreover, the N-terminal PTH form can be overproduced in some patients with parathyroid carcinoma and severe primary hyperparathyroidism, where it represents a much larger proportion of the circulating PTH immunoreactivity in the third-generation PTH assay. Nevertheless, the biological significance of the N-terminal PTH form is currently undetermined.

The bioactive PTH assay provides results that are approximately 50%–70% of those measured by the intact PTH assay in patients with CKD and approximately 15% lower than those in persons without CKD [63, 64]. There is evidence demonstrating that PTH values measured by the intact PTH assay and bioactive PTH assay are highly correlated. Nevertheless, the intact PTH assay is more available, more extensively validated, and less expensive than the bioactive PTH assay [66–68]. Therefore, the 2017 KDIGO CKD-MBD guideline suggested that the widely available second-generation PTH assays should continue to be used in routine clinical practice [61].  Table 1 summarizes the characteristics of the three different types of PTH assays.

## 5. Origin and Clinical Implications of Oxidized PTH in CKD

Patients with CKD are susceptible to intense oxidative stress, and this leads to extensive oxidative modification of protein structure. PTH can be oxidized on its two methionine residues at positions 8 and/or 18, and this results in the altered three-dimensional conformation of PTH [33]. In CKD patients, 70–90% of measured PTH are in the oxidized form [69]. Oxidized PTH binds poorly to PTHR1 and is unable to generate intracellular cAMP, and thus is biologically inactive in the animal models [33, 70, 71]. Intact and bioactive PTH assays measure both oxidized and nonoxidized PTH, resulting in higher PTH levels than when measured with a nonoxidized PTH assay. To measure the nonoxidized PTH, a serum sample pretreatment with the specific antibody targeted against the oxidized PTH is required prior to determination with a conventional PTH assay [69]. In addition, oxidized PTH can be precisely quantitated by the sensitive mass spectrometric assay.

Tepel et al. analyzed the association between nonoxidized PTH and mortality in hemodialysis patients and found that nonoxidized PTH was a good predictor of mortality [72]. However, the recent cohort study by Seiler-Mussler et al. demonstrated that the second-generation PTH assay was more closely associated with cardiovascular events, CKD progression, and all-cause mortality than nonoxidized PTH measurement in patients with CKD not receiving dialysis [73]. The unfavorable prognosis associated with PTH level measured by the intact PTH assay is hypothetically attributable to the oxidative stress status. However, the clinical implication of nonoxidized PTH measurement in CKD patients remains unresolved due to concerns regarding ex vivo oxidation of PTH [74]. Moreover, nonoxidized PTH target values for patients with CKD are still unknown and this warrants further research [75, 76].

## 6. Preanalytical Considerations for PTH Measurement

Preanalytical optimization, including specimen type, sampling time, sampling site, and storage condition, is crucial because PTH is relatively labile. PTH is generally measured in serum or preferably in ethylenediaminetetraacetic acid (EDTA) plasma because PTH is more stable in EDTA plasma than serum. It should be noted that PTH value in EDTA plasma tends to be up to 20% lower than that in serum from the same blood sample. Circulating PTH levels have a diurnal variation, and therefore, blood samples should preferably be obtained in the morning with an overnight fast. In hemodialysis patients, PTH concentration was higher (∼30%) in central blood (superior vena cava) than peripheral blood (antecubital vein) [77]. Thus, blood samples for PTH measurement should be collected from the same sample site (central or peripheral) for comparison both within and between individuals. PTH is stable in EDTA whole blood for at least 24 h at room temperature as compared to only 3 h in clotted whole blood. At 4°C, PTH is more stable in EDTA plasma (at least 72 h) than serum (at least 24 h), but should be frozen for longer storage [78]. Previous study suggested that PTH was more stable when measured by the third-generation rather than the second-generation PTH assay because of the instability of PTH fragments [79].

## 7. Current Guidelines for PTH Measurement in CKD-MBD and Future Perspective

The 2017 KDIGO guideline recommended monitoring serum calcium, phosphate, PTH, and alkaline phosphatase levels beginning in CKD G3a, and in CKD G4–G5D, ALP should be measured every 12 months, or more frequently in the presence of elevated PTH. In patients with CKD G4 and G5, the guidelines suggested that the monitoring intervals of serum PTH would be every 6–12 months and 3–6 months, respectively, or more frequently in the presence of elevated PTH. Notwithstanding, there is lack of randomized controlled trials to define an optimal PTH level for patients with CKD G3a-G5, clinical endpoints of hospitalization, fracture, and mortality. However, in clinical practice, the rising PTH levels in CKD G3a-G5 deserve assessment of modifiable factors, including vitamin D insufficiency or deficiency, hypocalcemia, hyperphosphatemia, and high phosphate intake.

The intact PTH assay is recommended as a standard PTH measurement in CKD-MBD patients. Bone biopsy is still the gold standard for the assessment of renal osteodystrophy. Although bone mineral density (BMD) by dual-energy X-ray absorptiometry (DXA) can predict fractures across the spectrum from CKD G3a to G5D, it does not distinguish among types of renal osteodystrophy [80–82]. Moreover, the use of bone turnover markers (BTMs) in CKD patients has been limited as many of these BTMs, such as procollagen type I N-terminal propeptide (PINP) and C-terminal telopeptide of type I collagen (CTX), are mainly excreted by the kidney; therefore, BTMs should be used with caution in patients with eGFR <30 mL/min/1.73 m^2^. In contrast, bone-specific alkaline phosphatase (BSAP, an osteoblast-derived bone formation marker) and tartrate-resistant acid phosphatase 5b (TRAP 5b, an osteoclast-derived bone resorption marker) are not affected by kidney function. However, unlike PTH, these BTMs are not routinely measured in patients with CKD due to a relatively high cost, limited availability of automated assays, and uncertain cut-offs for diagnosis of renal bone disease. Consequently, PTH remains the most commonly used surrogate marker in clinical prediction of renal osteodystrophy and fracture. However, previous cross-sectional studies provided conflicting results on the utility of biomarkers to predict underlying bone histology. This inconsistency is somewhat owing to the short half-lives of most of the circulating biomarkers (e.g., 2–4 minutes for PTH) and the long period of bone remodeling cycle (3–6 months). Therefore, the 2017 KDIGO CKD-MBD guideline suggests the continued use of trends in PTH rather than absolute “target” values for CKD-MBD management, and when trends in PTH are inconsistent, a bone biopsy should be considered.

Currently, the most commonly used assay for measuring PTH levels in clinical practice is the intact PTH assay. Although there is a good correlation between the intact and bioactive PTH assays in patients on maintenance dialysis, mean PTH levels are typically 30–50% lower in bioactive PTH assay than the intact PTH assay [63, 64]. This discrepancy in patients with CKD is due to the accumulation of non-(1-84) PTH fragments detected solely by the intact PTH assay.

Cinacalcet, a widely used oral calcimimetic agent, binds to the extracellular calcium-sensing receptor on parathyroid glands, then directly suppresses PTH production, and stimulates intraparathyroid PTH degradation to various PTH fragments. These effects result in a potential overestimation of intact PTH levels measured by the intact PTH assay [83]. However, the effect of cinacalcet on imprecision of serum PTH levels is controversial since the subsequent study in the larger hemodialysis patients demonstrated comparable accuracy of the intact PTH assay for monitoring PTH response to cinacalcet therapy compared with the bioactive PTH assay [84].

Because some of these non-(1-84) PTH fragments have biological effects distinct from that of PTH (1-84), an independent evaluation of PTH (1-84) and different forms of non-(1-84) PTH fragment may increase the diagnostic accuracy to predict the type of renal osteodystrophy in patients with CKD. Liquid chromatography coupled to tandem mass spectrometry (LC-MS/MS) in the clinical laboratory is engaging and is widely used in diagnostic laboratories for steroid hormones [85]. The enormous potential of LC-MS/MS already led to development of methods that are associated with immunoaffinity, in situ digestion, and mass spectrometry to improve an accurate discrimination and quantification of the circulating PTH forms [86, 87]. Further research is needed to address this interesting issue.

## 8. Conclusions

PTH regulates bone and mineral homeostasis in CKD. Therefore, an accurate measurement of PTH is crucial for clinical judgment of the physician. An intact PTH assay is a current standard measurement in general practice; however, it measures not only a full-length biologically active PTH (1-84) but also N-terminal truncated PTH fragments, a presumed PTH antagonist. Preanalytical conditions, including sampling and storage ambience, evidently affect the measurement of PTH. As a result of PTH assay variability, preanalytical sample errors, and the phenomenon of end-organ PTH hyporesponsiveness, the 2017 KDIGO CKD-MBD guideline recommended the broad range for serum PTH targets (2–9 times the upper normal limit of the PTH assay) in patients with CKD G5D in order to reduce the risk of inducing adynamic bone disease. However, a considerable proportion of CKD patients who have serum PTH levels within the recommended range still have renal osteodystrophy. These findings tend to arise due to the biological interference of PTH fragments and oxidized PTH which significantly accumulates in CKD patients. Therefore, the assessment of divergent biological effects of PTH fragments and the measurement of bioactive nonoxidized PTH could potentially be implemented for the precise management of CKD-MBD in the near future.

## Figures and Tables

**Table 1:** Comparison among the three generations of PTH assay.

	First-generation PTH assay	Second-generation PTH assay	Third-generation PTH assay
Name	C-terminal radioimmunoassay	Intact PTH assay	“Bioactive” or “biointact” or “whole” PTH assay

Technique	Single-antibody radioimmunoassay	Two-site sandwich immunoassay	Two-site sandwich immunoassay

Antibody	Detection antibody (diverse epitopes at the mid- or C-terminal part of the PTH)	Capture C-terminal antibody (amino acid positions 39-84) and detection N-terminal antibody (amino acid positions 12-18, 13-24, or 26-32)	Capture C-terminal antibody (amino acid positions 39-84) and detection N-terminal antibody (amino acid positions 1-4)

Percent of circulating PTH (1-84) in normal GFR (%)	20	80	95
Percent of circulating PTH (1-84) in CKD	5	50	85

Short C-terminal PTH fragment	Detectable	Not detected	Not detected

Long C-terminal PTH fragment or non-(1-84) PTH fragment	Detectable	Detectable	Not detected

N-terminal PTH form	Detectable	Not detected if the epitope is proximal (amino acid positions 12-18 or 13-24), detectable if the epitope is distal (amino acid positions 26-32)	Detectable

Current test availability	No	Yes (mostly in clinical practice)	Yes (mostly in research)

CKD, chronic kidney disease; GFR, glomerular filtration rate; PTH, parathyroid hormone.
